# Fluorescent Dihomooxacalix[4]arenes for the Detection of Nitroaromatic Compounds in Solution and in the Vapour Phase: Structural and Supramolecular Insights

**DOI:** 10.3390/molecules30193901

**Published:** 2025-09-27

**Authors:** Beatriz V. Gil, Alexandre S. Miranda, Paula M. Marcos, José R. Ascenso, Tiago Palmeira, Mário N. Berberan-Santos, Rachel Schurhammer, Neal Hickey, Siddharth Joshi, Silvano Geremia

**Affiliations:** 1 Centro de Química Estrutural, Institute of Molecular Sciences, Faculdade de Ciências, Universidade de Lisboa, Edifício C8, 1749-016 Lisboa, Portugal; beatriz.v.gil@gmail.com (B.V.G.); miranda.m.alexandre@gmail.com (A.S.M.); 2 Faculdade de Farmácia, Universidade de Lisboa, Av. Prof. Gama Pinto, 1649-003 Lisboa, Portugal; 3 Centro de Química Estrutural, Institute of Molecular Sciences, Instituto Superior Técnico, Universidade de Lisboa, Complexo I, Av. Rovisco Pais, 1049-001 Lisboa, Portugal; jose.ascenso@ist.utl.pt (J.R.A.); tiago.palmeira@tecnico.ulisboa.pt (T.P.); 4 IBB-Institute for Bioengineering and Biosciences, Instituto Superior Técnico, Universidade de Lisboa, 1049-001 Lisboa, Portugal; 5 Laboratoire de Modélisation et Simulations Moléculaires, Université de Strasbourg, UMR 7140, F-67000 Strasbourg, France; rschurhammer@unistra.fr; 6 Centre of Excellence in Biocrystallography, Department of Chemical and Pharmaceutical Sciences, University of Trieste, via L. Giorgieri 1, 34127 Trieste, Italy; nhickey@units.it (N.H.); siddharth.joshi@units.it (S.J.); sgeremia@units.it (S.G.)

**Keywords:** dihomooxacalix[4]arenes, nitroaromatic compounds sensing, X-ray diffraction, fluorescence quenching, NMR studies, DFT calculations

## Abstract

Two fluorescent ureido-dihomooxacalix[4]arene derivatives containing naphthyl residues at the lower rim (**1** and **2**) were studied for the detection of nitroaromatic compounds (NACs) in solution and in vapour phases. Their affinity in solution was determined by UV-Vis absorption, fluorescence and NMR spectroscopy. For NAC vapour sensing, calixarenes were dispersed in a polytetrafluoroethylene (PTFE) matrix. Four new solvated crystals of dihomooxacalix[4]arene **2** were obtained and the solvent’s influence on its structural characteristics was investigated. The solvent-dependent structural variations observed in the crystal structures highlight the intrinsic flexibility of the calixarene framework. Such conformational adaptability, evident in the disruption and reorganization of hydrogen bonding and π–π interactions, is directly relevant to nitroaromatic sensing, where a rapid and reversible host response is crucial for effective detection. Theoretical calculations were also performed to provide further insights on the binding process. The corrected Stern–Volmer constants (*K*_SV_) obtained showed that both receptors present selectivity for TNP and follow the same quenching order (TNP > NT > NB > DNT > TNT > DNB). Factors other than electron density distribution should dominate the quenching extent and therefore the values of the SV constants, which will be greatly overestimated if no correction to the inner filter effect is applied. Detection of NB and NT and vapours by both calixarenes produced a complete, very fast (2 to 5 s), and reversible quenching, indicating the potential use of this porous PTFE–calixarene matrix for the sensing of volatile NACs.

## 1. Introduction

Nitroaromatic compounds (NACs), considered as environmental pollutants and toxic to living organisms, are found in soils and groundwaters due to their extensive use in industries, such as agrochemical, dye, and pharmaceutical. They can originate health problems in animals and human beings, including skin irritation, anemia, cataracts, and kidney and liver damage, among other diseases, and even after degradation, their by-products are still powerful carcinogenic agents [1,2,3]. In addition, nitroaromatic compounds such as trinitrotoluene, dinitrotoluene, and trinitrophenol are common explosives used for military purposes and are the major components of unexploded landmines. Nowadays, explosives continue to be a threat to international security, and thus their detection remains a crucial mission for all the countries in their antiterrorist activities. These compounds have a vast destruction potential and can be inexpensively and readily prepared from online protocols [1,2,3].

Several methods have been developed for trace detection of explosives, including gas chromatography–mass spectrometry (GC-MS), high-pressure liquid chromatography (HPLC), surface-enhanced Raman spectroscopy, nuclear quadruple resonance, ion mobility spectrometry, and cyclic voltammetry, among others [4]. Due to the very low vapour pressure of explosives, real-time monitorization of the gas and liquid phases with the techniques previously described is difficult, owing to the need for expensive and complex equipment with poor sensitivity and lack of portability. Fluorescence spectroscopy presents advantages over the former techniques owing to its extreme sensitivity, high stability and portability, fast response, ease of handling and low cost [5].

Numerous fluorescent sensors have been developed for explosive monitorization in the solid, liquid and vapour phases [6,7]. The interaction between analytes and probes causes variations (increase or decrease) in the fluorescence intensity, lifetimes, wavelengths and anisotropy, which can be used to detect explosives. Calixarenes are among these fluorescence-based sensing materials and have been broadly explored in various areas [8,9,10,11]. These macrocyclic compounds have well-organized cavities available in different sizes and conformations, and their upper and lower rims can be chemically modified, resulting in a very large number of derivatives [12,13]. Fluorogenic units such as naphthalene, anthracene, pyrene, naphthalimide, dansyl and coumarin have been included in the calixarene skeleton, giving rise to potential fluorescent probes for NACs [14,15,16,17,18,19,20]. Calixarene moieties have also been incorporated in fluorescent conjugated polymers and used in explosive detection [21,22,23,24]. Homooxacalixarenes, calixarene analogues in which the CH_2_ bridges are partly or completely replaced by CH_2_OCH_2_ groups [25,26], in particular hexahomotrioxacalix[3]arenes, have already been investigated to detect explosives [27,28,29].

**Scheme 1:**
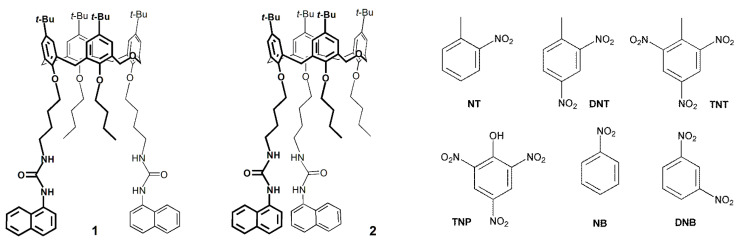
Chemical structures of receptors **1** and **2** and of the NACs studied.

As part of our ongoing investigation on fluorescent homooxacalixarene-based receptors for the recognition of relevant ionic species [30,31,32], we have recently extended our research to the detection of nitroaromatic compounds (explosives) by dihomooxacalix[4]arene derivatives. Thus, in this paper are reported the recognition properties of two cone lower rim naphthylurea-dihomooxacalix[4]arene receptors (**1** and **2**) towards the NACs 2-nitrotoluene (NT), 2,4-dinitrotoluene (DNT), 2,4,6-trinitrotoluene (TNT), 2,4,6-trinitrophenol (TNP), 1-nitrobenzene (NB) and 1,3-dinitrobenzene (DNB) (Scheme 1). Their sensing ability in solution was assessed by UV-Vis, fluorescence, and proton NMR spectroscopy. The detection of NB and NT vapours was also carried out. Single-crystal X-ray diffraction analysis of four new solvated crystals of dihomooxacalix[4]arene **2** are presented to investigate the influence of solvents on the structural characteristics, while computational studies were performed to provide deeper understanding of the binding process.

## 2. Results and Discussion

### 2.1. Single-Crystal X-Ray Diffraction Studies

Many crystallization trials of dihomooxacalix[4]arene naphthylurea **2** with two nitroaromatic compounds (TNP and DNB molecules) were performed by the evaporation method in several solvents or solvent mixtures (CH_2_Cl_2_ + EtOH, *n*-hexane, MeCN, MeOH + CHCl_3_, DMSO, DMF, THF, TFE, xylene). However, no nitroaromatic guests were found in any determined structures. On the other hand, in addition to our previously published structure, **2α** [31], which did not contain co-crystallized solvent molecules, four new solvated pseudo-polymorphs, **2β**, **2γ**, **2δ**, and **2ε**, were obtained during these crystallization trials (Table 1). The failure to obtain crystals with incorporated NACs can be rationalized by several factors. First, the relatively weak host–guest interactions between the calixarene cavity and NAC were insufficient to stabilize the guest in the solid state during crystallization. Second, crystal formation depends not only on the strength of host–guest association but also on the relative solubility of the free host and the host–guest complex, which in turn is governed by crystal packing preferences. Solvent molecules, present in large excess and often better suited to lattice inclusion, were therefore preferentially incorporated.

**Figure 1:**
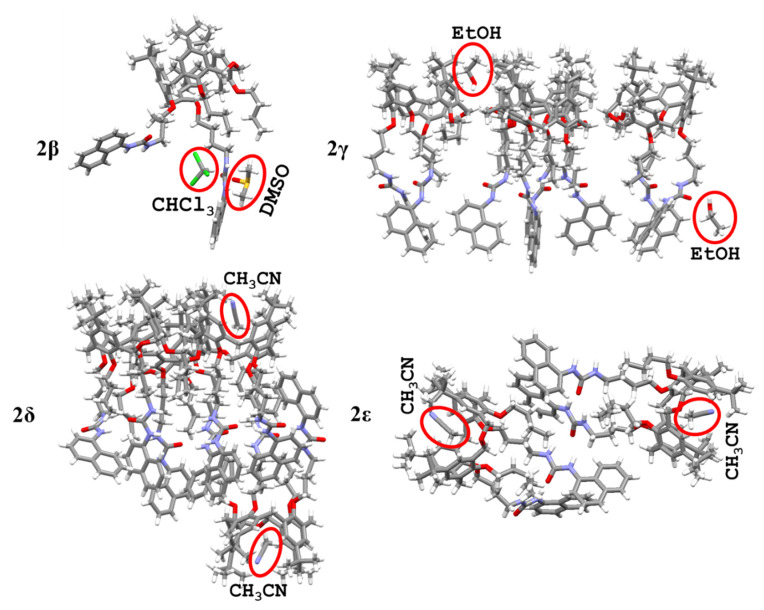
Stick representation of the crystallographic independent molecules found in the four new pseudo-polymorphs of **2**. Atomic species are represented in CPK colors. The co-crystallized solvent molecules are highlighted with red circles. The disorder groups have been omitted for clarity.

The monoclinic centrosymmetric **2β** structure was obtained in the presence of a DMSO/CHCl_3_ mixture of solvents. The asymmetric unit is composed of one molecule of **2** in pinched cone conformation, one DMSO molecule and one CHCl_3_ molecule (Figure 1). The DMSO is involved, as an acceptor, in a bifurcated H-bond with one urea group (N···O distances of 2.850 Å and 2.920 Å). In addition, the O-atom of the sulfoxide group is involved in a weak H-bond with the CHCl_3_ (C···O distances of 3.108 Å). The unsolvated form, **2α**, shows intramolecular and intermolecular H-bonds involving the urea groups, thereby forming continuous 1D chains (Figure S1a) [31]. The crystal packing is characterized by inversion-related antiparallel polymeric chains of these bifurcated H-bonds. In the **2β** form, the co-crystallized solvents completely interrupt the intramolecular H-bonds and interfere with the intermolecular H-bond network. This results in a reorganization of the H-bonds and very different conformations of the naphthyl arms (Figure 1). In each molecule, the two divergent naphthyl arms are involved in different H-bond interactions. The first ureido group, involved as an H-bond donor with DMSO, forms an intermolecular bifurcated H-bond with two NH functions of the second urea of an adjacent molecule. The C=O group of this second urea is not involved in any significant H-bond interactions. This produces the propagation of polymer-like chains characterized by a single intermolecular bifurcated H-bond link between successive molecules (Figure S1b).

The triclinic centrosymmetric **2γ** form was obtained from an ethanol/CH_2_Cl_2_ solvent mixture. The asymmetric unit is composed of four molecules of **2** in pinched cone conformation and one and a half co-crystallized ethanol molecules (Figure 1). These solvent molecules are located in interstitial sites of the packing of **2** and they are not involved in H-bonds. The H-bond network of the urea groups, which form a chain of molecules in which the cups of adjacent molecules are oriented in opposite directions, is very similar to that observed for the unsolvated **2α** form, also obtained from the same ethanol/CH_2_Cl_2_ solvent mixture [31]. However, the two structures differ in the crystal packing organization in terms of the mutual orientation of the 1D chains. In particular, while in the **2α** form the 1D chains with the closest H-bond network are all parallel (Figure S2a), in the **2γ** form there are consecutive couples of parallel and antiparallel chains (Figure S2b).

The triclinic centrosymmetric **2δ** form was obtained in the presence of acetonitrile. The asymmetric unit consists of four molecules of **2** in the pinched cone conformation and two acetonitrile molecules hosted in the cups of two of these four independent molecules (Figure 1). This represents an unusual crystal structure in which both the complexed and uncomplexed forms of the dihomooxacalix[4]arene coexist within the same crystal. In this case as well, the co-crystallized solvent, which is held inside the host cavity by the well-known CH-π interactions between the methyl group and the aromatic rings, does not interfere with the ureido H-bond network and the crystal packing is similar to that observed for **2γ** (Figure S2b).

The triclinic non-centrosymmetric **2ε** form was also obtained in the presence of acetonitrile. The asymmetric unit consists of two host–guest complexes of **2** with acetonitrile (Figure 1). Both crystallographically independent complexes are involved in the H-bond network, forming the same 1D chain. Similarly to the other structures, the 1D chain is organized with alternating orientations of the cups; however, in this case, the crystal packing is characterized by all parallel 1D chains (Figure S2c). As a result, a polar crystal has been obtained, with all the H-bonds oriented in the same direction.

Interestingly, the intramolecular H-bond with one ureido group as donor and one as acceptor (or intermolecular H-bonds in **2β**) generates an inherent chirality in the otherwise symmetric derivative **2**. In the case of **2ε**, the two crystallographically independent molecules are enantiomers in terms of this inherent chirality, in which one (enantiomer M) has the H-bond donor in position 4 (H-bond acceptor in 3), while the other (enantiomer P) has the H-bond donor in position 3 (H-bond acceptor in 4). Therefore, all the centrosymmetric structures, as well as this non-centrosymmetric one, are composed of racemic mixtures of inherently chiral enantiomeric pairs (Figure S3). In addition to this chirality, due to the intramolecular bifurcated H-bond, there is also the asymmetry of the cone conformation with respect to a regular C_s_ symmetric molecule, which has already been observed in various dihomooxacalix[4]arene derivatives [31,33,34]. In fact, this asymmetry is due to a typical pinched cone conformation observed in calix[4]arenes; however, in dihomooxacalix[4]arenes, the oxa bridge results in a conformation chirality in which the pinched aryl group linked by the oxa bridge is located to the left (or to the right) of this bridge when viewed from above the top rim (Figure S3). Considering the H-bond M enantiomers, the canting angle (Table 2) between the aryl plane and the mean plane of the bridging methylene carbon atoms is systematically larger when the ureido substituent on the lower rim of the calixarene cone acts as a hydrogen bond acceptor (position C, which ranges from 130 to 153°) compared to when it acts as a hydrogen bond donor (position D, which ranges from 92 to 103°). Angles greater than 90° indicate that the *tert*-butyl groups on the upper rims lean outwards from the center of the cone. The two canting angles involving the aryl groups bridged by the oxo moiety are also very different (position A, which ranges from 108 to 125° and position B, which ranges from 71 to 97°). Therefore, the pinched aryl groups are always those in positions B and D. The insertion of the acetonitrile guest has a significant effect on the opening of the cup, although the pinched aryl groups remain B and D. The canting angle of B is the most affected (Table 2), with a change of about 20°, becoming outward oriented with respect to the inward orientation of the guest-free molecules. Therefore, the presence of the guest results in a more symmetric pinched conformation of the cone with respect to the conformation in the absence of the guest (Table 2). It is important to note that the cone conformation of the calixarene influences the relative position of the substituents on the lower rim [35]. However, the presence of a long aliphatic chain used as a spacer between the cone and the ureido function can overcome this geometric restriction. In fact, the intramolecular H-bonds are not significantly affected. It is interesting to note that in all eleven crystallographically independent molecules of **2** with intramolecular H-bonds, the pinched ureido-aryl group is always the one in which the NH groups act as intramolecular H-bond donors. Similarly, in the **2β** structure without intramolecular H-bonds, the pinched ureido-aryl group is the one in which the NH groups act as intermolecular bifurcated H-bond donors to a ureido group of an adjacent molecule.

The mutual orientation of the naphthyl moieties is almost perpendicular in all the eleven crystallographically independent molecules with intramolecular H-bonds (dihedral angles between naphthyl mean planes range from 64 to 88°, Table 2). In fact, a typical edge-to-face π-stacking arrangement is always present, with the formation of a CH-π intramolecular interaction between the hydrogen atom in position 7 of one naphthyl and the aromatic face of the other naphthyl moiety. Interestingly, this aromatic face is always located on the arm that contains the C=O of the ureido group involved in the intramolecular bifurcated H-bond.

Overall, this crystal structure investigation suggests that the ureido groups have a very strong tendency to form an extensive H-bond network incorporating intramolecular bifurcated H-bonds. The intramolecular H-bond creates a distinction between the two ureido arms of **2** (one acting as an H-bond donor and the other as an acceptor), which generates an element of inherent chirality. In addition, the pinched cone conformation, in conjunction with the dihomooxa bridge, introduces another element of inherent chirality in the solid state. Interestingly, these two elements of chirality appear to be correlated, as in all twelve crystallographically independent molecules characterized in the five pseudo-polymorphs, only one of the two possible enantiomeric couples was observed (Figure S3). Furthermore, except for **2β**, these molecules are characterized by a classical intramolecular edge-to-face π-stacking arrangement of the two naphthyl groups. This interaction consistently involves a CH-π contact between the H-7 atom of the naphthyl ring of the arm bearing the ureido group acting as an H-bond donor and the aromatic face of the naphthyl ring on the arm with the ureido group acting as an H-bond acceptor. These structural features remain consistent in the unsolvated form **2α**, the ethanol-solvated form **2γ**, and the host–guest complexes with acetonitrile **2δ** and **2ε**. However, the solvated form **2β**, crystallized with DMSO and chloroform, stands out as an exception. The co-crystallized DMSO interferes with the H-bond network, disrupting the intramolecular H-bond and resulting in two divergent naphthyl arms. This effect was not observed for ethanol, which can act as both an H-bond donor and acceptor, nor with acetonitrile, which is capable of forming specific host–guest complexes. In the latter case, the change in cup conformation due to solvent inclusion does not significantly alter the intramolecular and intermolecular interactions involving the lower rim of **2**. The different behavior observed could have some implication on the capacity of the studied calixarenes for the detection of nitroaromatic compounds in different solvents.

### 2.2. Detection of Nitroaromatic Compounds

#### 2.2.1. UV-Vis Absorption and Fluorescence Studies in Solution

The potential of asymmetric (**1**) and symmetric (**2**) dihomooxacalix[4]arene naphthyl ureas, previously obtained in the cone conformation [31], as receptors to detect nitroaromatic compounds was investigated by fluorescence titrations in dichloromethane.

Both receptors display absorption and emission bands centered around 285 nm and 380 nm in dichloromethane, respectively, the latter band being specific to the naphthyl urea group.

**Figure 2:**
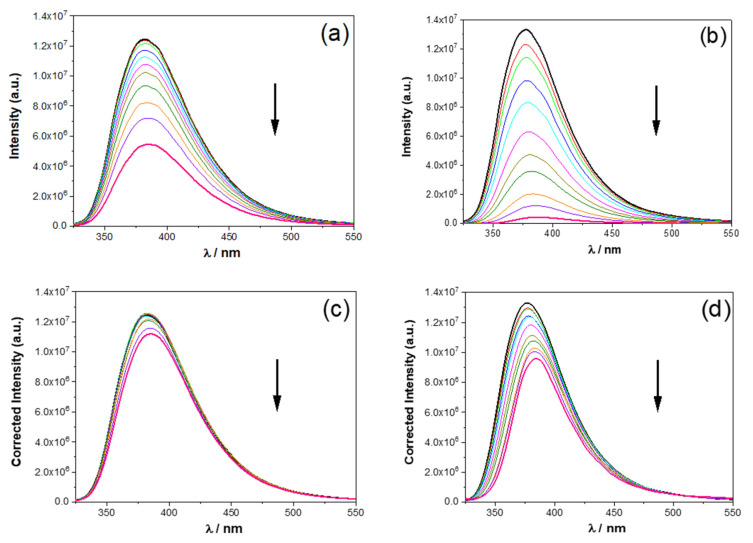
Changes in the emission spectra of (**a**) **1** + TNT, (**b**) **2** + TNP, before correction; (**c**) **1** + TNT, (**d**) **2** + TNP, after correction. The arrows indicate the effect of increasing amounts of the NAC. [**1**] = [**2**] = 1.0 × 10^−5^ M in CH_2_Cl_2_; λ_ex_ = 285 nm.

The addition of all NACs (up to 30 equiv.) to compounds **1** and **2** caused a significant decrease in the fluorescence intensity, as shown in Figure 2a,b. As the NACs studied in this work significantly absorb at 285 nm (Figure S4), the excitation wavelength used, it is necessary to correct the inner filter effect (absorption of the NAC at the excitation wavelength). This was carried out as previously described [28], leading to corrected emission spectra (Figure 2c,d). Correction of the excitation inner filter effect was carried out using Equation (1):

(1) $$F_{corr}=10^{\Delta A/2}F$$

where *F_corr_* is the corrected fluorescence intensity, *F* the measured fluorescence intensity, and Δ*A* = *A* − *A*_0_, where *A* and *A*_0_ are the absorbances (for 1 cm pathlength) at the excitation wavelength for the solutions of the calixarenes with and without NACs, respectively [28]. Quenching is still observed, but to a much lesser extent. Unfortunately, this correction is frequently ignored in complexation studies based on fluorescence, resulting in overestimated equilibrium constants.

The fluorescence quenching of the electron-rich aromatic fluorophore moieties present in calixarenes **1** and **2** involves interaction of the naphthyl groups with the electron-deficient NACs. The fluorescence decays of **1** and **2** can only be fitted with a sum of 3 or 4 exponentials (see Table 3), with a dominant long component of 11–12 ns. As shown in Table 3, the fluorescence lifetimes of the naphthyl fluorophores show minor changes upon complexation, pointing to an essentially static quenching mechanism (formation of a charge-transfer ground state complex), as observed with related systems [29].

**Figure 3:**
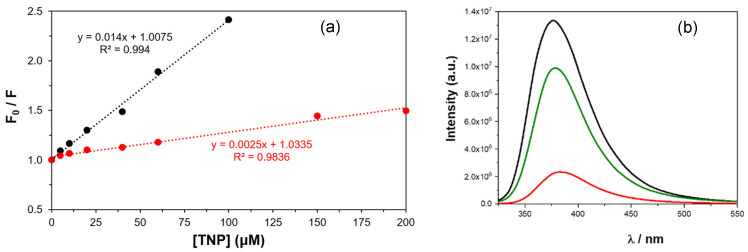
(**a**) Stern–Volmer uncorrected (black circles) and corrected (red circles) plots of **2** upon addition of TNP. For clarity’s sake, points (black circles) above 10 equiv. were omitted. (**b**) Fluorescence spectra of **2** alone (black) and in the presence of 20 equiv. of TNP, before (red) and after (green) correction for the inner filter effect. [**2**] = 1.0 × 10^−5^ M in CH_2_Cl_2_; λ_ex_ = 285 nm.

Apparent and corrected Stern–Volmer constants (*K*_SV_) were calculated from the slopes of uncorrected and corrected fluorescence intensity versus [NAC] plots, as illustrated in Figure 3a,b for compound **2** with TNP, for which uncorrected and corrected *K*_SV_ are 14,000 M^−1^ and 2500 M^−1^, respectively. The corrected *K*_SV_ constants are presented in Table 4 along with the corresponding detection limits. The absolute values of the *K*_SV_ also lend credence to a static mechanism [1], as they would imply quenching rate constants higher than diffusion control (1.5 × 10^10^ M^−1^ s^−1^ in dichloromethane at room temperature) by up to one order of magnitude (e.g., 2.6 × 10^11^ M^−1^ s^−1^ for compound **1** with TNP), if dynamic quenching were operative (in such a case, *k_q_* = *K*_SV_/τ_0_ where τ_0_ is the fluorescence lifetime in the absence of NACs; see Table 3). The raw (uncorrected) data deviate from linearity for high concentrations, but linearity is restored upon correction.

**Figure 4:**
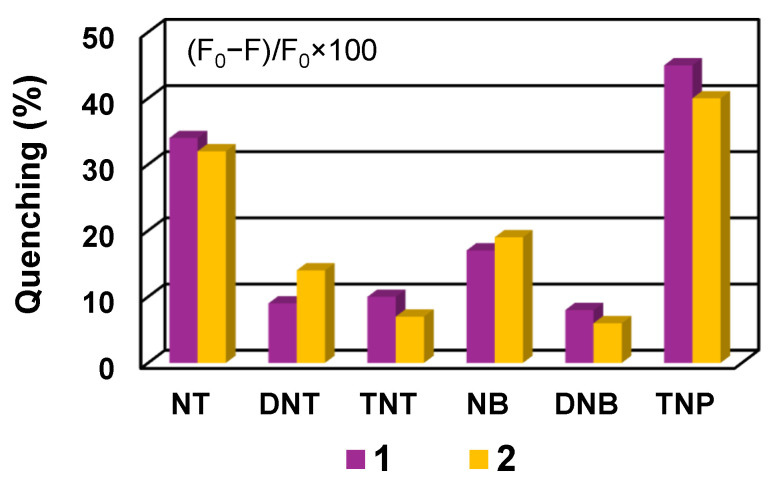
Fluorescence quenching of **1** and **2** after addition of 30 equiv. of NACs. [**1**] = [**2**] = 1.0 × 10^−5^ M in CH_2_Cl_2_; λ_ex_ = 285 nm.

As mentioned before, quenching of the fluorescence intensity was observed for both **1** and **2** with all NACs (Figure 4, Figures S5 and S6). From the analysis of the data, it can be seen that both calixarenes follow the same quenching order (TNP > NT > NB > DNT > TNT > DNB), and **1** seems to be slightly more affected than **2**. This quenching trend correlates well with the Stern–Volmer constants and the limits of detection (Table 4). The greatest efficiency was obtained for the highest electron-deficient nitroaromatic compound, TNP (*K*_SV_ = 3000 and 2500 M^−1^, respectively), which exhibited the lowest detection limits (18 and 21 µM, respectively). The affinity order for **1** and **2** found within each NAC group (NT > DNT > TNT and NB > DNB) seems to indicate that factors other than electron density distribution control the quenching extent and consequently the magnitude of the SV constants.

Comparing our results with those reported in the literature concerning a calix[4]arene bearing four ethynyl naphthyl groups at the upper rim, it turns out that this compound shows a very strong affinity for *p*-nitrophenol (*K*_SV_ = 10060 M^−1^ in MeCN), followed by TNP and DNT and, to a much lesser extent, by TNT (*p*-NP >> TNP > DNT >> TNT). According to the authors, the highest fluorescence quenching caused by *p*-NP is due not only to its electron affinity but also to “host–guest interaction by matching size” [17]. In contrast, two hexahomotrioxacalix[3]arenes bearing pyrene-linked triazole residues at their lower [27] or upper [29] rims were reported and exhibited the greatest quenching efficiency for TNP (~70 or 90%, respectively), followed by *p*-NP (~35%), TNT (~20–25%), and DNT (~10–15%). Despite the high detection capacity of calixarenes for NACs, the reasons for their selectivity towards a specific guest are not obvious, and the understanding of all host–guest interactions involved is not totally clear either [9].

#### 2.2.2. Quenching Studies with NAC Vapours

Direct sensing of NAC vapours is of great interest, as this method is more adequate for their detection in the environment and for security applications. NB and NT were the selected NACs for a vapour study. NB and NT are liquids at room temperature, with vapour pressures of about 0.1 mmHg [36] (corresponding to a concentration of ca. 5 × 10^−6^ M). On the other hand, at room temperature, TNP and TNT are solids with very low vapour pressures [37,38] and could not be detected.

**Figure 5:**
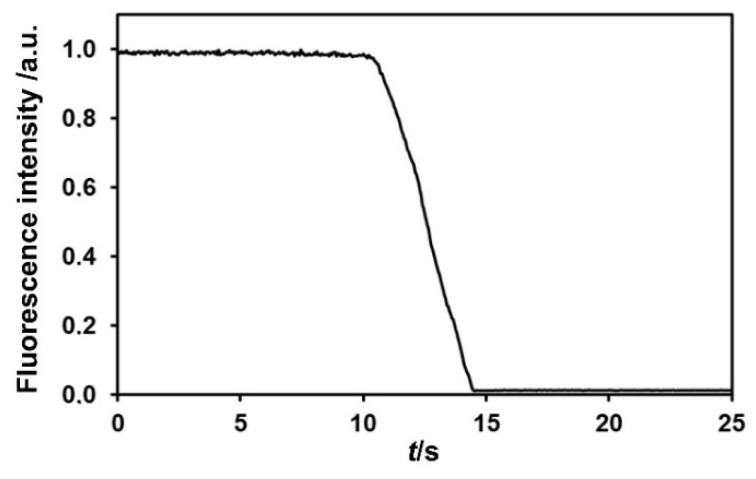
Quenching of **2** in a PTFE matrix by NT vapour at room temperature.

Dihomooxacalix[4]arenes **1** and **2** were dispersed in a porous polytetrafluoroethylene (PTFE) matrix (180 µm thickness). After irradiation of the film for several seconds to establish the initial fluorescence intensity (100%), a drop of NAC liquid (NB and NT) was quickly deposited at the bottom of the cell. Following this, with both NB and NT, there is fast (within 2 to 5 s) and complete fluorescence quenching (Figure 5 and Figure S7) at room temperature. The duration of the fast fluorescence decrease period (2–5 s) appears to be mainly controlled by the time needed to attain liquid–vapor equilibrium in the cell. The quenching was observed to be fully reversible. Both calixarenes displayed the same behavior towards both NACs.

The obtained results open the possibility of sensing volatile NACs (and other volatile analytes) using a porous PTFE–calixarene matrix (never tried before to the best of our knowledge), which is inexpensive, simple to prepare, effective, stable, and inert, and allows fast sensing of vapours, comparing advantageously with other systems mentioned in the literature [2,24].

#### 2.2.3. NMR Studies

**Figure 6:**
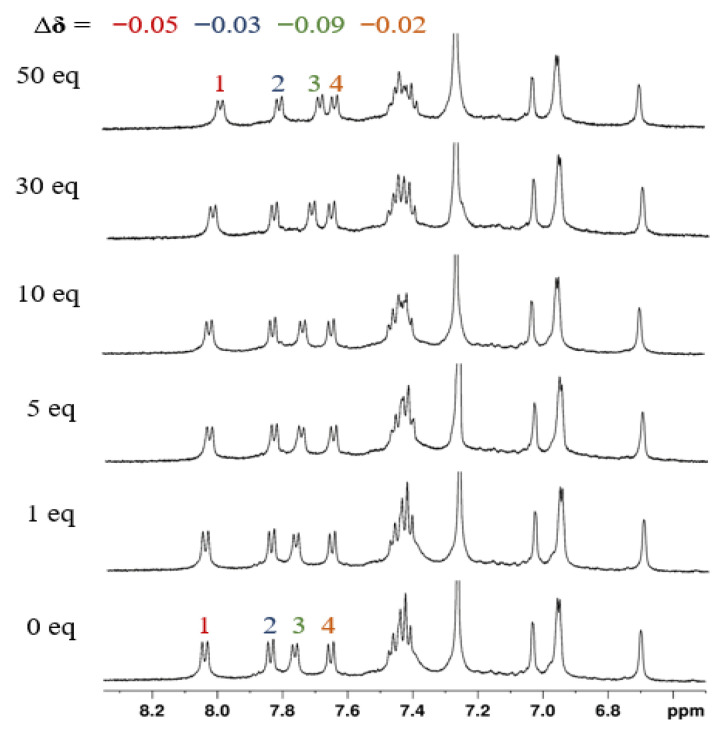
Partial ^1^H NMR spectra (500 MHz, CDCl_3_, 25 °C) of naphthyl urea **2** with several equiv. of TNP.

The interaction between receptors **1** and **2** and two representative nitroaromatic explosives, TNP and DNB, was also carried out by proton NMR titrations. Increasing amounts of the NACs, up to 50 equivalents, were added to CDCl_3_ solutions of receptors **1** and **2** and showed that both receptors have some affinity for TNP. In the case of the asymmetric derivative **1**, extensive peak overlapping is present in the spectra, which makes it difficult to follow them during the titrations. However, for receptor **2**, as shown in Figure 6, small upfield shift variations (Δ*δ* ≤ 0.09 ppm) for the naphthyl peaks can be observed upon addition of 50 equiv. of TNP. This shielding effect may reflect π-π stacking of the naphthyl fluorophore with the nitroaromatic group of TNP and the corresponding ring current effects. Similar results were reported in the literature with related calix[4]arenes [14,16,18]. No changes occurred in the NMR spectra of both receptors over the titrations with DNB (Figure S8). All these findings corroborated the previous fluorescence results.

#### 2.2.4. Theoretical Calculations

**Figure 7:**
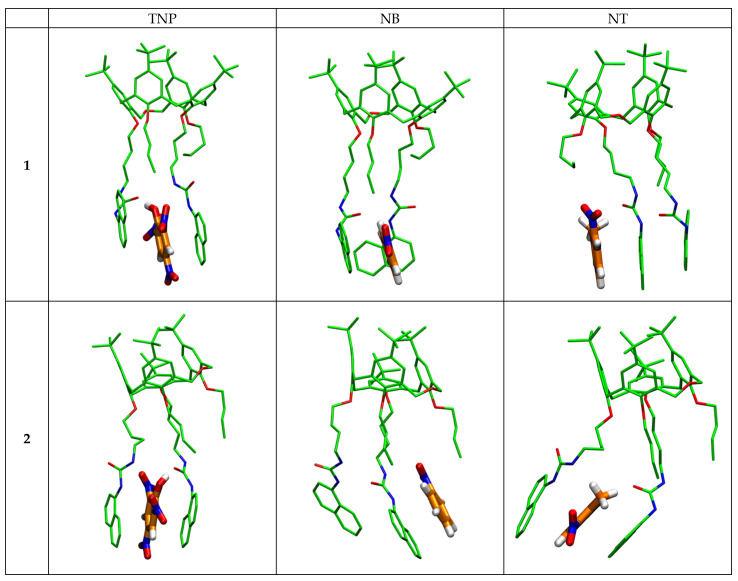
Final structures of the NAC–calixarene complexes after QM optimization.

To further investigate the relative stability, the interaction energies and the structures of the complexes between calixarenes **1** and **2** and the nitroaromatic compounds TNP, NB, and NT, quantum mechanical calculations were performed. Different starting positions of the guests were tested, either positioning them between the calixarene arms or outside. Table 5 presents the host–guest interaction energies in kJ mol^−1^ (total energies in hartree of the isolated molecules and of the most stable complexes are given in Tables S1 and S2), and Figure 7 illustrates the structures of the optimized complexes.

In general, ∆*E* host–guest interaction energies follow the Stern–Volmer constants and the quenching extent. All the NAC guests interact through π∙∙∙π stacking with the terminal naphthyl groups of the hosts. Hydrogen bond interactions between the urea groups are maintained in almost all cases, which stabilize the structures formed. In the case of TNP, the most energetically favorable position is observed for the guest positioned between the two naphthyl arms of the host (“sandwich” type). The strongest interactions were obtained with this NAC and both calixarenes (−109.33 and −92.09 kJ mol^−1^ for **2** and **1**, respectively). Interaction energies are lower for NB and NT, and the most stable form is not necessarily the “sandwich” type. NB guest interacts via π∙∙∙π stacking with only one naphthyl group and better with calixarene **2** than with calixarene **1** (−73.72 vs. −42.59 kJ mol^−1^) and keeps the hydrogen bonds between the urea groups. In the case of NT, the molecule interacts more favorably with **1** than with **2** (−56.23 vs. −36.99 kJ mol^−1^), either by interacting with only one naphthyl arm, maintaining the intramolecular hydrogen bonding between the urea groups (calixarene **1**), or via the “sandwich” type (calixarene **2**), but in this case disrupting the interactions between the urea groups and destabilizing the calixarene.

The variation in the H-bond distances found for the host–guest interactions of calixarenes **1** and **2** with the NACs also supports the results obtained. In the case of **2**∙∙∙NT, the interaction distances between the oxygen atom of the carbonyl group of one urea residue and the NH groups of the other are 5.890 Å and 6.174 Å, respectively, which can no longer be considered as hydrogen bonds. For **2**∙∙∙NB, the two urea residues of the calixarene interact with each other through both CO∙∙∙NH bonds, with distances of 1.863 Å and 2.058 Å, respectively. In the case of **2**∙∙∙TNP, the structure contains a loose CO∙∙∙NH hydrogen bond of 3.903 Å and a short CO∙∙∙HO_TNP_ (1.476 Å). Regarding calixarene **1**, due to a more regular cone conformation where the pendant arms are facing each other, the urea∙∙∙urea interactions are shorter than those for calixarene **2**, which presents a distorted cone conformation, probably resulting from some constraint between the adjacent arms. For the three NACs, these intramolecular interactions are maintained in addition to the **1**∙∙∙guest interaction. For NT and NB, the CO∙∙∙NH hydrogen bonds are strong and short (1.917 Å/1.973 Å and 1.915 Å/2.031 Å, respectively). The case of TNP is slightly different, as both of its OH and nitro groups interact with the urea carbonyl function and the NH group, respectively. Thus, for this guest, the calixarene∙∙∙NAC interaction is based on three hydrogen bonds: CO∙∙∙HO (1.472 Å), NH∙∙∙ONO (2.078 Å) and CO∙∙∙HN (2.654 Å).

**Figure 8:**
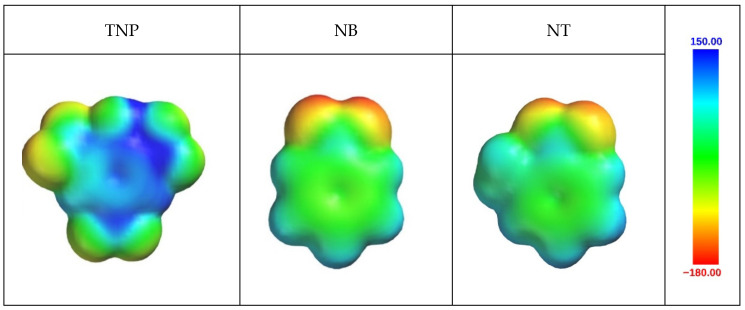
Electrostatic potential surface maps (in kcal·mol^−1^) computed (B3LYP/6-31G*) with Spartan’24 (Spartan 24, Wavefunction Inc., Irvine, CA, USA) for different NACs.

Electrostatic potential surfaces of the different guests (Figure 8) show differences that may explain the observed results. The phenyl group of the NAC is more positive for the best bound TNP, while it is around zero for NT and NB. Large differences in guests’ polarity also influence the type of interaction they have with the hosts. This agrees with the interaction energies obtained by QM calculations.

## 3. Materials and Methods

### 3.1. Determination of Crystallographic Structures of ***2***

The evaporation method was applied for the crystallization of dihomooxacalix[4]arene naphthylurea **2** with two nitroaromatic compounds (TNP and DNB molecules) using several solvents or solvent mixtures (CH_2_Cl_2_ + ETOH, *n*-hexane, MECN, MEOH + CHCl_3_, DMSO, DMF, THF, TFE). Initially, near-saturated solutions of the macrocycle were prepared in suitable solvents. Followed by the covering of vials using perforated caps and finally, placed in a crystallization room with a controlled temperature of 18 °C. Crystals grew within a few hours to days. The single-crystal X-ray diffraction data collection was conducted at the XRD1 beamline of the Elettra synchrotron, Trieste, Italy. The rotating-crystal method was employed, utilizing a Dectris Pilatus 2M area detector (Baden-Daettwil, Switzerland) and monochromatic radiation with a wavelength of 0.700 Å. Single crystals were mounted on a nylon loop after being dipped in paratone cryoprotectant and flash-frozen under a nitrogen stream at 100 K. Diffraction data were indexed and integrated using the XDS package [39], while scaling was carried out with XSCALE [40]. Structures were solved using the SHELXT program [41], and the refinement was performed with SHELXL [42] by the full-matrix least-squares (FMLS) method on F^2^. For the refinement, non-hydrogen atoms were anisotropically refined with the exception of some disordered groups with low occupancy factors, which were refined isotropically (Figure S9). The hydrogen atoms, located on different Fourier maps, were added at the calculated positions and refined using the riding model. The occupancy factors of the disordered atoms were initially refined and, after a few cycles, were fixed on the basis of these refinements and on the evaluation of their thermal motions.

The monoclinic centrosymmetric **2β** structure crystallized from CHCl_3_/*n*-hexane solvent mixture after recycling an unsuccessful crystallization trial of **2** with TNP in DMSO/xylene solution. The asymmetric unit is composed of one molecule of **2** in cone conformation, one DMSO molecule and one CHCl_3_ molecule. Both solvent molecules have two position disorder with a total partial occupancy of 0.85 (DMSO, 0.70/0.15) and 0.65 (CHCl_3_, 0.50/0.15). The triclinic centrosymmetric **2γ** form was obtained from ethanol/CH_2_Cl_2_ solvent mixture in presence of **2** and DNB. The asymmetric unit is composed of four molecules of **2** in cone conformation, one and half co-crystallized solvent molecules. One ethanol molecule shows a two-position disorder, with 0.5/0.5 occupancy factors. The triclinic centrosymmetric **2γ** form was obtained from ethanol/CH_2_Cl_2_ solvent mixture in presence of **2** and DNB. The asymmetric unit is composed of four molecules of **2** in cone conformation and one and half co-crystallized solvent molecules. One ethanol molecule shows a two-position disorder, with 0.5/0.5 occupancy factors. The triclinic centrosymmetric **2γ** form was obtained from ethanol/CH_2_Cl_2_ solvent mixture in presence of **2** and DNB. The asymmetric unit is composed of four molecules of **2** in cone conformation and one and half co-crystallized solvent molecules. One ethanol molecule shows a two-position disorder, with 0.5/0.5 occupancy factors. The triclinic non-centrosymmetric **2ε** form was also obtained from acetonitrile/CH_2_Cl_2_ mixture of **2** with DNB. The asymmetric unit is composed of two host–guest complexes of **2** with acetonitrile. The summary of crystal data and the refinement details with structural information are presented in Table S3, while the contents of the unit cells are illustrated in Figure S10 and the solvent-accessible volumes of the four structures are illustrated in Figure S11.

### 3.2. UV-Vis Absorption and Fluorescence Studies

Absorption and fluorescence studies were carried out using a Shimadzu UV-3101PC UV-Vis-NIR spectrophotometer (Kyoto, Japan) and a Horiba Jobin Yvon Fluorolog 3-22 iHR fluorometer (Kyoto, Japan) in right-angle geometry (except when otherwise noted), respectively. The studies were made in CH_2_Cl_2_ at 25 °C. The absorption spectra were recorded between 200 and 400 nm and the emission ones between 320 and 550 nm using quartz cells with an optical path length of 1 cm. The excitation wavelengths used were at the maximum absorption of the calixarenes. The titrations were performed cell by cell with different concentrations of nitroaromatic compounds up to 30 equiv. and with a constant concentration of the receptors (10 μM). Emission spectra were corrected for the spectral response of the optics and the photomultiplier. In addition, the emission spectra were further corrected for the internal filter effect. Time-resolved fluorescence intensity decays were obtained using the single-photon timing method with laser excitation and microchannel plate detection, with the setup already described [43]. The excitation wavelength used was at the maximum absorption of the calixarene, and the emission wavelength was at the maximum emission. For the NAC vapor studies, calixarenes were dispersed in porous polytetrafluoroethylene (PTFE) solid matrices (180 μm thickness), obtained by pressing PTFE powder (1 μm particles) mixed with the calixarenes. Fluorescence measurements were in this case made in the front-face geometry. Decay data analysis with a sum of exponentials was achieved by means of a Microsoft Excel spreadsheet specially designed for lifetime analysis that considers the convolution with the IRF.

### 3.3. 1H NMR Studies

Several aliquots (up to 50 equiv.) of the nitroaromatic compounds (TNP and DNB) in CDCl_3_ were added to CDCl_3_ solutions of the hosts (1.25 × 10^−3^ M) directly in the NMR tube. The spectra were recorded on a Bruker Avance III 500 spectrometer (Billerica, MA, USA) after each addition of the NACs, and the temperature of the NMR probe was kept constant at 25 °C.

### 3.4. DFT Calculations

Stationary points were optimized with the Gaussian 09 program [44] with B3LYP [45] functions and with the 6-31G(d,p) basis set. A D3-Grimme correction [46] was also used, and dichloromethane solvent was considered with the PCM model. Experimental X-ray diffraction structure determinations of the calixarene receptors were employed as the starting structures for geometry optimization. All reported structures were confirmed as energy minima, with no negative eigenvalue in the Hessian matrix.

## 4. Conclusions

The ability of two cone dihomooxacalix[4]arene derivatives, doubly functionalized on the lower rim with naphthyl urea moieties in distal and proximal positions (**1** and **2**, respectively), to be used as fluorescent sensors for the detection of nitroaromatic compounds was investigated by UV-Vis, fluorescence and NMR studies in solution. A static fluorescence quenching mechanism was observed, and the corrected Stern–Volmer constants showed that both compounds have selectivity for TNP and follow the same quenching order (TNP > NT > NB > DNT > TNT > DNB). Factors other than electron density distribution rule the quenching extent and consequently the magnitude of the SV constants, which would be greatly overestimated if no correction to the inner filter effect was applied. DFT calculations corroborated the experimental results. Sensing of NAC vapours was also performed by dispersing the calixarenes in a porous polytetrafluoroethylene (PTFE) matrix. Detection of NB and NT vapours by both dihomooxa compounds produced a complete, very fast (2 to 5 s), and reversible quenching. These results point to the possibility of sensing volatile NACs using an inexpensive, stable, and inert porous PTFE–calixarene matrix, never tried before to the best of our knowledge.

Despite numerous attempts, no nitroaromatic guests were found in any determined structures. Nevertheless, our solution- and gas-phase studies provide strong evidence for binding and reveal the dynamic, reversible nature of the interaction. The inability to trap the complex in the solid state may reflect the labile yet strong interactions that are crucial for the rapid response and reversibility observed in the sensing experiments.

Four new solvated crystals of **2** were obtained and the impact of solvents on its structural characteristics was examined. The crystal structure analysis reveals that the ureido-functionalized dihomooxacalix[4]arene adopts a consistent intramolecular H-bonding pattern and edge-to-face π-stacking arrangement of the naphthyl groups across multiple pseudo-polymorphs. These interactions give rise to two correlated elements of inherent chirality. Solvent inclusion generally preserves these structural features, except in the DMSO/chloroform-solvated form **2β**, where DMSO disrupts the H-bond network, leading to conformational divergence of the naphthyl arms. These observations are directly relevant to sensing, since the detection of NACs relies on the ability of the host to reorganize in response to external stimuli. The disruption of the intramolecular H-bonding network by DMSO, for example, illustrates how small changes in the environment can induce significant conformational variation. Analogously, NAC binding in solution or in the gas phase is expected to exploit this structural adaptability, resulting in a rapid and reversible sensing response.

To find out how to improve the NAC’s detection by these macrocycles, homooxacalixarenes bearing different fluorogenic units (such as anthracene or pyrene) through shorter and less flexible spacers are currently under investigation.

## Figures and Tables

**Table 1:** Unit cell parameters, space group, and number of crystallographic independent molecules of **2**; Z’ of the pseudo-polymorphs obtained with various co-crystallized solvent molecules.

	Co-Crystallized Solvent	Unit Cell						S.G.	Z’
		a (Å)	b (Å)	c (Å)	α (°)	β (°)	γ (°)		
**2α** *	—	11.473	55.43	12.189	90	102.08	90	P2_1_/n	1
**2β**	DMSO/CHCl_3_	20.486	16.422	24.136	90	100.186	90	P2_1_/c	1
**2γ**	EtOH	18.218	27.893	30.697	93.026	92.572	91.321	P-1	4
**2δ**	CH_3_CN	18.199	30.879	30.919	63.535	87.731	89.789	P-1	4
**2ε**	CH_3_CN	11.411	12.425	27.438	84.719	83.052	83.765	P1	2

* Structure published in ref. [31].

**Table 2:** Comparison of cone conformations and mutual orientation of naphthyl groups in the pseudo-polymorphs of **2**: dihedral angles between corresponding aryl planes of the calixarene cones (A, B, C, and D shown in Figure S3) and the mean planes of the bridging methylene carbon atoms of dihomooxacalix[4]arenes and dihedral angles between the two naphthyl mean planes (Nap-Nap).

Molecule	A (°)	B (°)	C (°)	D (°)	Nap-Nap (°)
**2α**	125	83	140	92	88
**2β**	116	71	130	100	57
**2γ**-1	120	73	151	98	87
**2γ**-2	117	76	144	101	75
**2γ**-3	120	74	147	96	81
**2γ**-4	118	76	153	100	86
**2δ**-1	119	74	153	98	84
**2δ**-2@CH_3_CN	117	93	141	101	64
**2δ**-3@CH_3_CN	108	95	138	103	88
**2δ**-4	119	73	145	95	75
**2ε**-1@CH_3_CN *	118	97	137	98	80
**2ε**-2@CH_3_CN	118	97	135	99	78

* The labeling scheme of the aryl groups is inverted with respect to the others.

**Table 3:** Three- and four-exponential analysis of fluorescence decays of **1** and **2** with 30 equiv. of NACs in CH_2_Cl_2_ at 25 °C.

	τ_1_/ns (%)	τ_2_/ns (%)	τ_3_/ns (%)	τ_4_/ns (%)	$\bar{\tau }$/ns
** 1 ** *	0.27 (9)	2.21 (13)	11.49 (78)	—	9.4
** 1 + NT **	0.27 (9)	2.14 (14)	9.43 (77)	—	7.6
** 1 + DNT **	0.28 (9)	2.19 (14)	10.11 (77)	—	8.1
** 1 + TNT **	0.27 (10)	2.02 (12)	9.97 (78)	—	8.0
** 1 + NB **	0.25 (10)	2.04 (12)	9.54 (78)	—	7.7
** 1 + DNB **	0.26 (10)	2.04 (11)	10.69 (79)	—	8.7
** 1 + TNP **	0.27 (10)	2.01 (14)	10.84 (77)	—	8.6
** 2 ** *	0.20 (6)	0.99 (7)	3.77 (31)	11.96 (56)	7.9
** 2 + NT **	0.20 (7)	0.77 (6)	3.35 (28)	9.86 (59)	6.7
** 2 + DNT **	0.24 (10)	—	2.00 (12)	9.52 (78)	7.7
** 2 + TNT **	0.23 (7)	1.23 (8)	3.83 (38)	11.17 (47)	6.8
** 2 + NB **	0.20 (6)	0.75 (6)	3.09 (35)	9.46 (53)	6.1
** 2 + DNB **	0.21 (7)	0.81 (6)	3.36 (34)	10.45 (53)	6.8
** 2 + TNP **	0.21 (7)	0.94 (7)	3.51 (34)	10.43 (52)	6.7

* [**1**] = [**2**] = 1.0 × 10^−5^ M; λ_ex_ = 285 nm.

**Table 4:** Corrected Stern–Volmer constants (K_SV_/M^−1^) and detection limits (DL/μM) * of **1** and **2** (1.0 × 10^−5^ M) in CH_2_Cl_2_ at 25 °C.

		NT	DNT	TNT	NB	DNB	TNP
**1**	*K_SV_*	1550	490	440	1200	270	3000
	DL	31	105	153	52	212	18
**2**	*K_SV_*	1200	465	320	1000	150	2500
	DL	39	135	189	48	214	21

* For a 5% decrease in fluorescence intensity.

**Table 5:** Host–guest interaction energies (∆*E* in kJ mol^−1^).

	∆*E* (kJ mol^−1^)	
	1	2
TNP	−92.09	−109.33
NB	−42.59	−73.72
NT	−56.23	−36.99

## Data Availability

The original contributions presented in this study are included in the article and Supplementary Materials. Further inquiries can be directed to the corresponding authors.

## Supplementary Materials

The following supporting information can be downloaded at: https://www.mdpi.com/article/10.3390/molecules30193901/s1, crystallographic data; electronic absorption spectra of the NACs in CH_2_Cl_2_; fluorescence intensity plots; fluorescence quenching by NAC vapours; NMR titration spectra; DFT calculations.
