# Supplementary material for: Fluorescent Dihomooxacalix[4]arenes for the Detection of Nitroaromatic Compounds in Solution and in the Vapour Phase: Structural and Supramolecular Insights
Source: Molecules. 2025 Sep 27;30(19):3901. doi: 10.3390/molecules30193901 (PMC12525786; doi:10.3390/molecules30193901)
Supplement: Supplementary file 1 [file molecules-30-03901-s001.zip › molecules-3817823-supplementary.pdf]

Supporting Information

Fluorescent Dihomooxacalix[4]arenes for the Detection of Nitroaromatic Compounds in Solution and in the Vapour Phase. Structural and Supramolecular Insights

Beatriz V. Gil, Alexandre S. Miranda, Paula M. Marcos,* José R. Ascenso,
Tiago Palmeira, Mário N. Berberan-Santos,* Rachel Schurhammer,
Neal Hickey, Siddharth Joshi, Silvano Geremia

List of contents

1. X-ray crystal structures of **2**: 1D polymeric chains and crystal packing representations 2
2. Stick representation of the enantiomeric couples found in all the structures of **2** 4
3. Electronic absorption spectra of the NACs in CH_2Cl_2 4
4. Fluorescence intensity plots 5
5. Time evolution of fluorescence of **1** and **2** in a PTFE matrix upon exposure to NAC vapours at room temperature 7
6. RMN titration spectra 8
7. DFT calculations 8
8. Crystal data refinement details 9
9. ORTEP drawings, contents of the unit cells and solvent-accessible volumes of the four structures of **2** 10

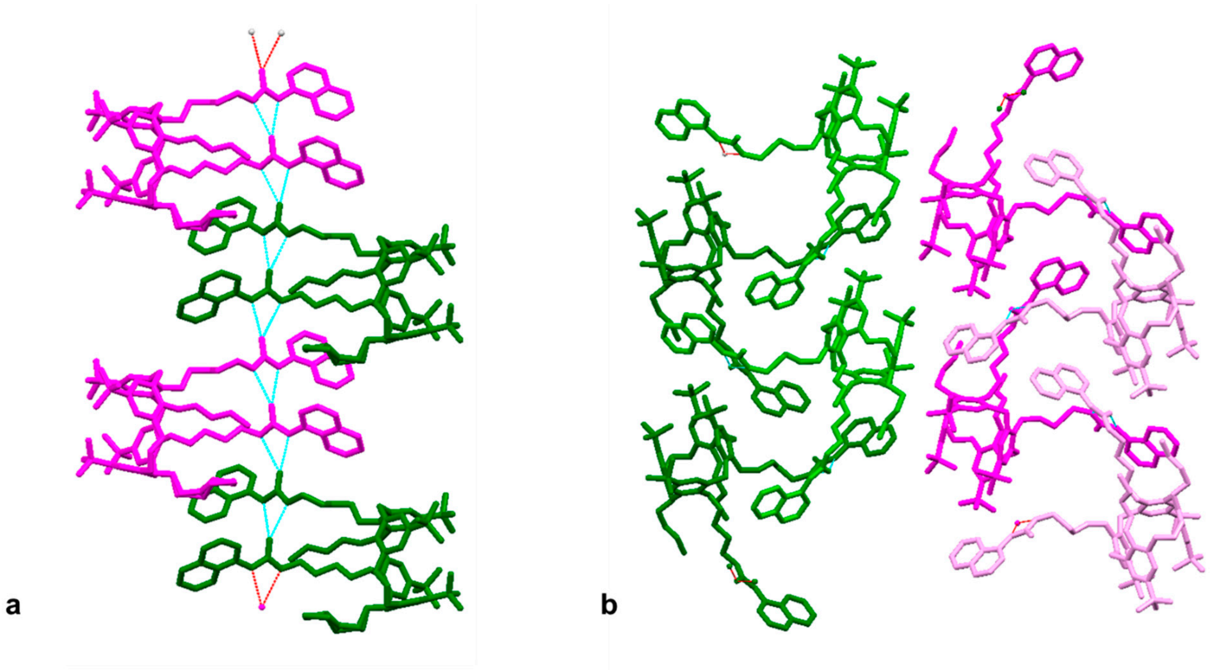

Figure S1. 1D polymeric chains observed in the crystal structures of **2**. a) Row of molecules interconnected by bifurcated H-bonds, observed in all structures except for **2β**; b) Enantiomeric helices of molecules interconnected by intermolecular H-bonds, as observed in **2β**. The different colours represent molecules with opposite inherent chirality: green = M and violet = P.

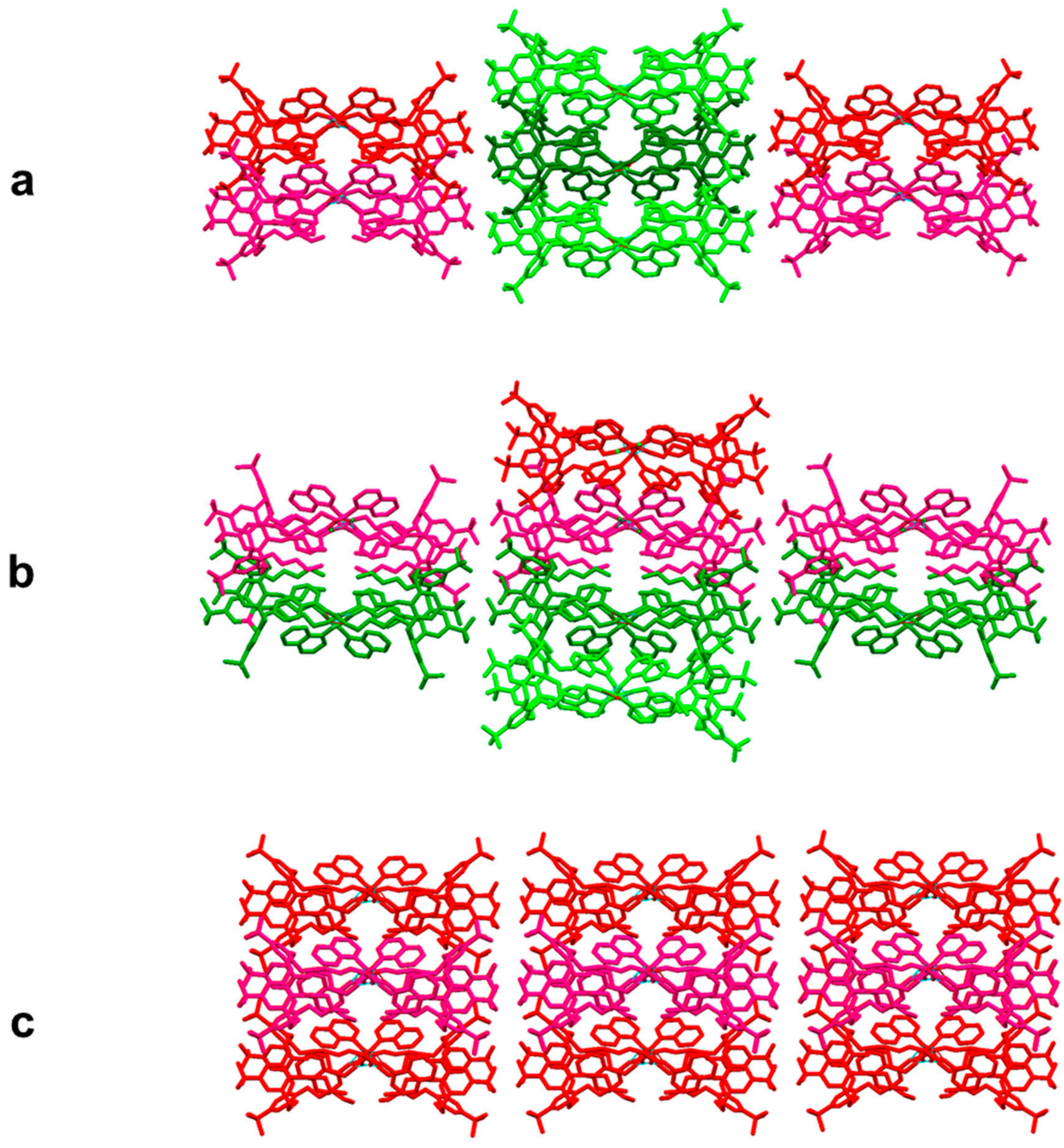

Figure S2. Crystal packing representation of the structures of **2** viewed along the H-bond network. a) Packing of the 1D chains as observed in the **2α** form; b) Packing of the 1D chains as observed in the **2γ** and **2δ**; c) Packing of the 1D chains as observed in the **2ε** form. Different colours represent parallel and antiparallel chains.

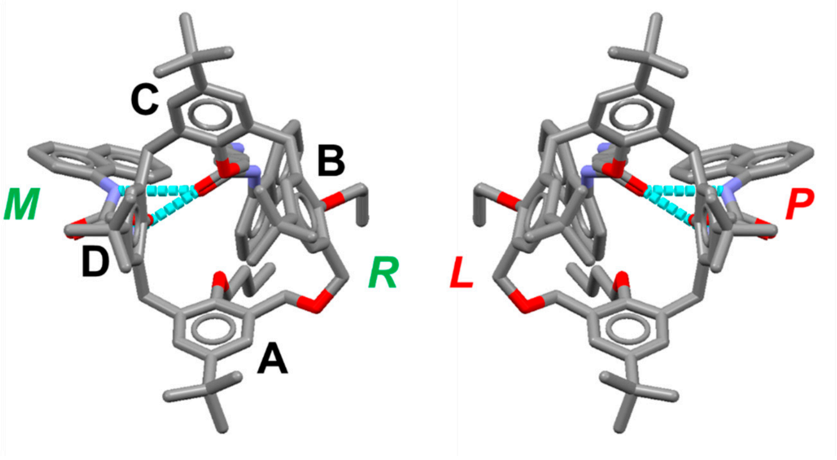

Figure S3. Stick representation of the enantiomeric pairs found in all the crystal structures of **2**, showing both types of inherent chirality: M/P arising from the position of the H-bond donor/acceptor and R/L resulting from the pinched-cone shape and the position of the dihomooxa bridge. The labelling scheme for the position of the aryl rings, as used in Table 2, is also shown.

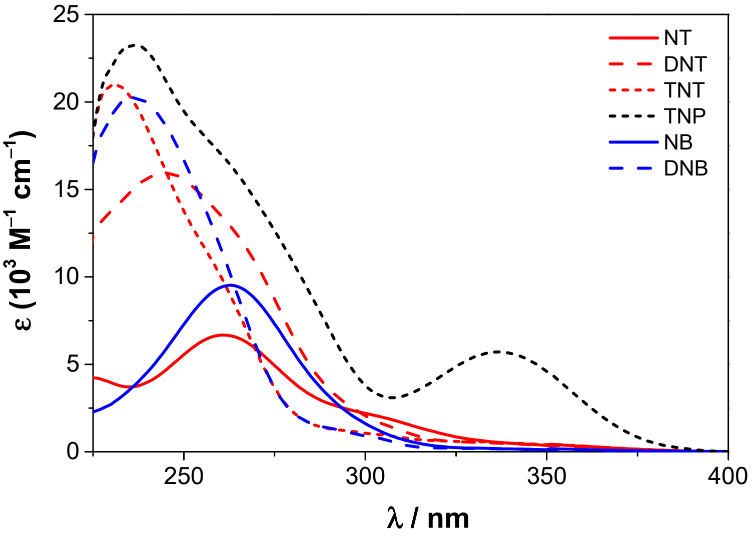

Figure S4. Electronic absorption spectra of the NACs in CH_2Cl_2.

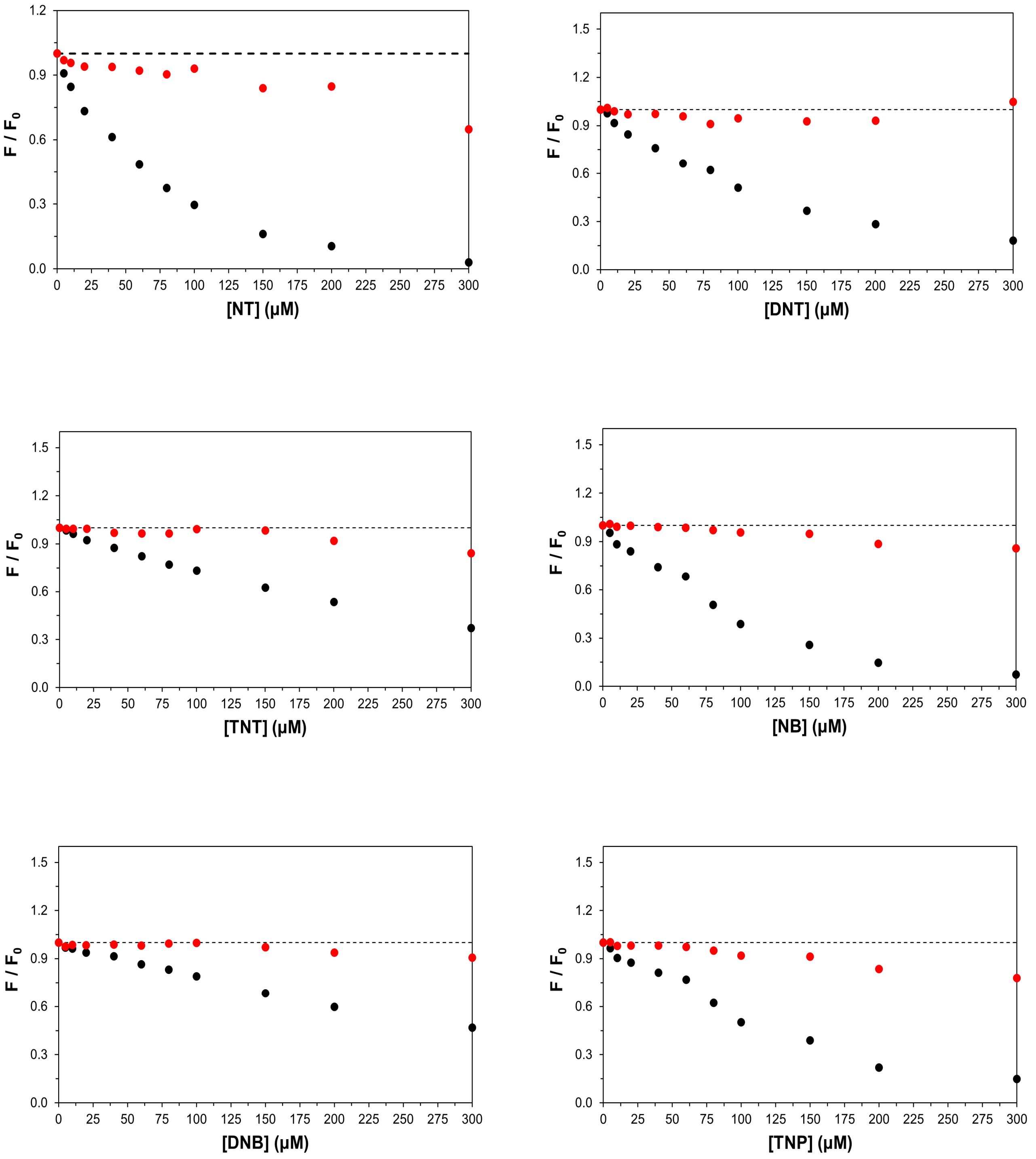

Figure S5. Uncorrected relative fluorescence intensity (black circles) and corrected (red circles) for the inner filter effect of Napht urea **1** upon addition of the NACs (up to 30 equiv) in CH_2Cl_2. F_0 is the intensity in the absence of quencher and F the intensity in the presence of the NAC. [**1**] = 1.0×10^{-5} M; λ_{ex} = 285 nm.

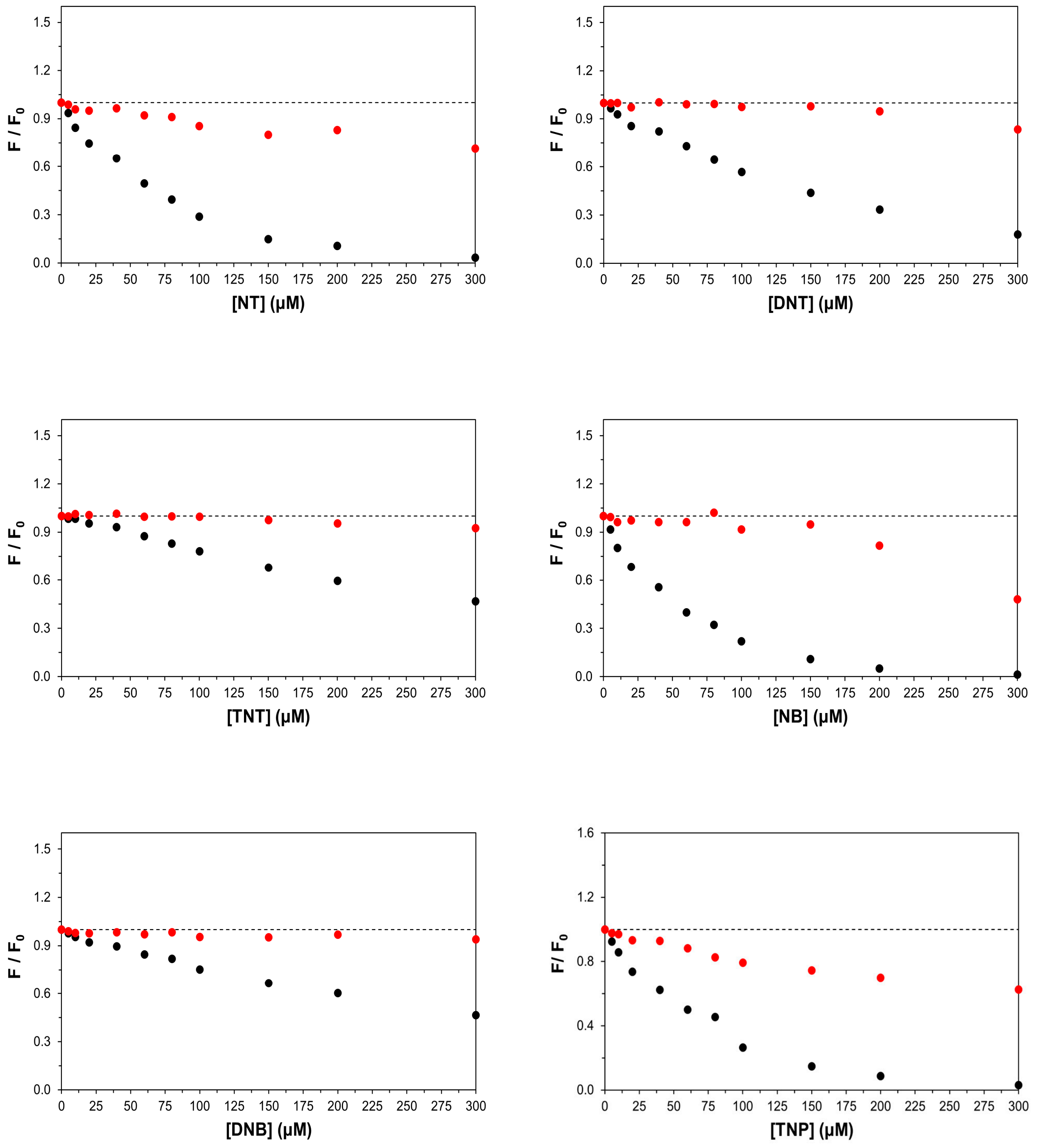

Figure S6. Uncorrected relative fluorescence intensity (black circles) and corrected (red circles) for the inner filter effect of Napht urea **2** upon addition of the NACs (up to 30 equiv) in CH_2Cl_2. F_0 is the intensity in the absence of quencher and F the intensity in the presence of the NAC. [**2**] = 1.0×10^{-5} M; λ_{ex} = 285 nm.

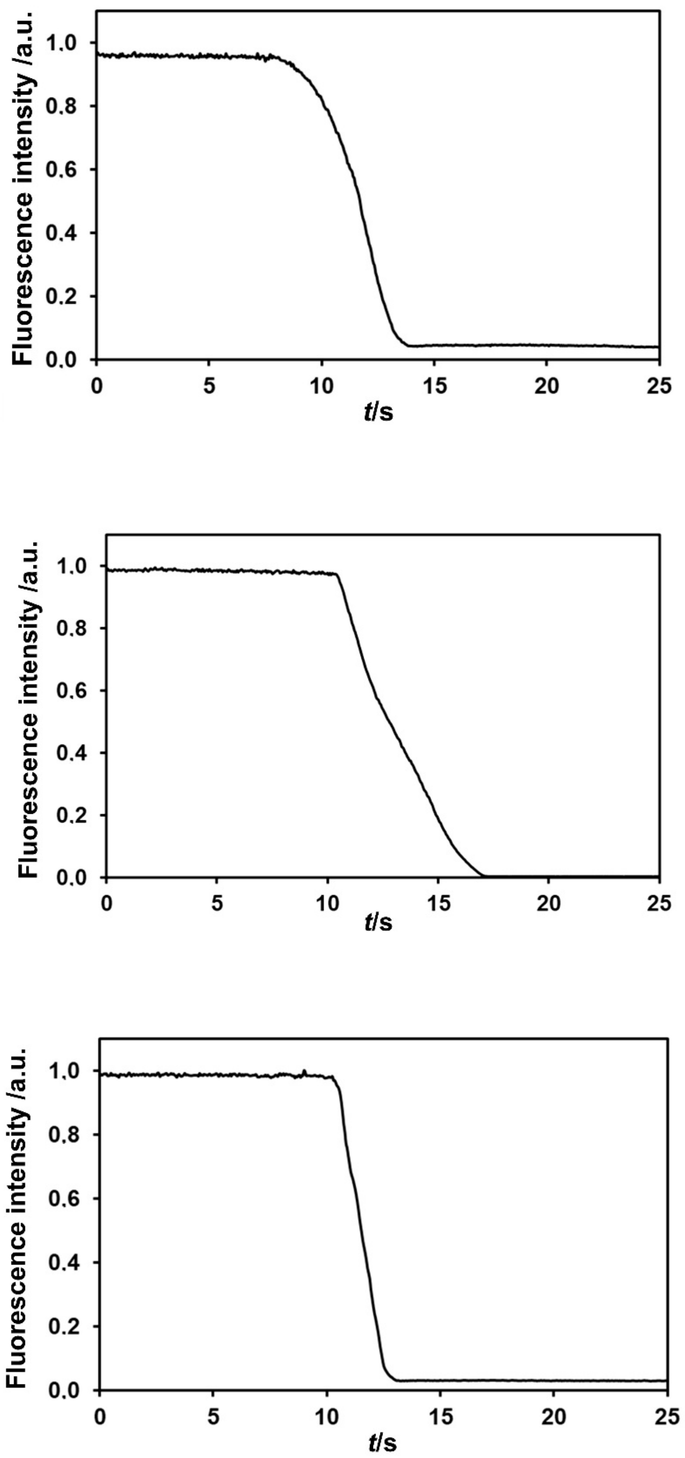

Figure S7. Time evolution of fluorescence of **1** and **2** in a PTFE matrix upon exposure to NAC vapours at room temperature. The initial intensity decrease coincides with the introduction of a drop of liquid NAC in the cell. From top to bottom: **1** + NB, **1** + NT and **2** + NB. For **2** + NT see Figure 5.

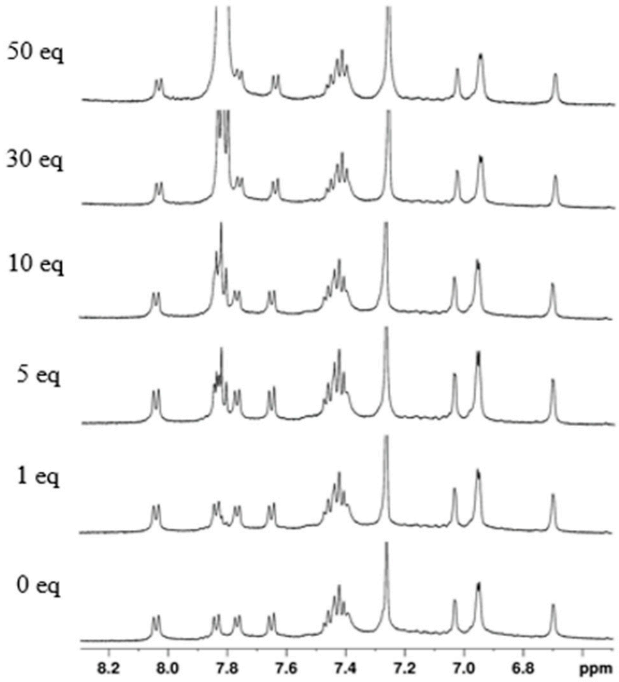

Figure S8. Partial ^{1}H NMR spectra (500 MHz, $CDCl_3$, 25 °C) of naphthyl urea **2** with several equiv of DNB.

Table S1. QM total energy (in hartree) of the isolated studied molecules

**1**	−3973.046205
**2**	−3973.043026
TNP	−921.015926
NB	−436.789207
NT	−476.112254

Table S2. QM total energy (in hartree) and calixarene···guest interaction energy (in $kJ.mol^{-1}$)

	TNP	NB	NT	Δ*E*(TNP)	Δ*E*(NB)	Δ*E*(NT)
**1**	−4894.0972	−4409.8516	−4449.1799	−92.09	−42.59	−56.23
**2**	−4894.1006	−4409.8603	−4449.1694	−109.33	−73.72	−36.99

Table S3. Summary of the crystal data refinements of the four new pseudo-polymorphic forms of **2**

	2β	2γ	2δ	2ε
Empirical formula	$C_{83}H_{106}N_4O_7$ 0.85(C_2H_6SO) 0.65($CHCl_3$)	$C_{83}H_{106}N_4O_7$.0.375(C_2H_6O)	$C_{83}H_{106}N_4O_7$.0.5(C_2H_3N)	$C_{83}H_{106}N_4O_7$.C_2H_3N
Formula weight	1415.71	1288.99	1292.24	1312.77
Temperature (K)	100(2)	100(2)	100(2)	100(2)
Wavelength (Å)	0.7	0.7	0.7	0.7
Crystal system	Monoclinic	Triclinic	Triclinic	Triclinic
Space group	P 2_1/c	P -1	P -1	P 1
Unit cell Dimensions (Å. °)	a = 20.486(4)<br>b = 16.422(2)<br>c = 24.136(6)<br>α = 90<br>β = 100.19(7)<br>γ = 90	a = 18.218(8)<br>b = 27.893(12)<br>c = 30.697(12)<br>α = 93.026(13)<br>β = 92.572(15)<br>γ = 91.32(3)	a = 18.199(17)<br>b = 30.879(18)<br>c = 30.92(2)<br>α = 63.535(13)<br>β = 87.73(3)<br>γ = 89.79(2)	a = 11.411(4)<br>b = 12.4250(17)<br>c = 27.438(8)<br>α = 84.72(2)<br>β = 83.052(18)<br>γ = 83.76(4)
Volume (Å^3)	7992(3)	3391.1(15)	15541(20)	3826.5(18)
2 theta range °	1.575 – 25.940	0.947 – 21.687	1.079 – 24.394	1.477 – 25.980
Z	4	8	8	2
ρ_{calcd} (g/cm^3)	1.177	1.101	1.105	1.139
μ (mm^{-1})	0.150	0.067	0.067	0.069
F(000)	3046	5582	5592	1420
Reflections collected	105431	241928	338665	90968
Independent reflections	16282	37000	52002	29723
Data parameters/ restraints	973/837	3422/4733	3441/6	1775/2403
GOOF	1.030	1.087	1.076	1.024
Final *R* indices [I>2σ(I)]	R_1 = 0.0971<br>wR_2 = 0.2739	R_1 = 0.1233<br>wR_2 = 0.3017	R_1 = 0.1059<br>wR_2 = 0.2806	R_1 = 0.1097<br>wR_2 = 0.2662
R indices (all data)	R_1 = 0.1414<br>wR_2 = 0.3142	R_1 = 0.1883<br>wR_2 = 0.3590	R_1 = 0.1713<br>wR_2 = 0.3270	R_1 = 0.1482<br>wR_2 = 0.2939
Max. Diff. peak (e Å^{-3})	0.806/-0.606	0.716/-0.564	0.849/-0.514	0.791/-0.423
CCDC code	24755535	24755536	24755537	24755538

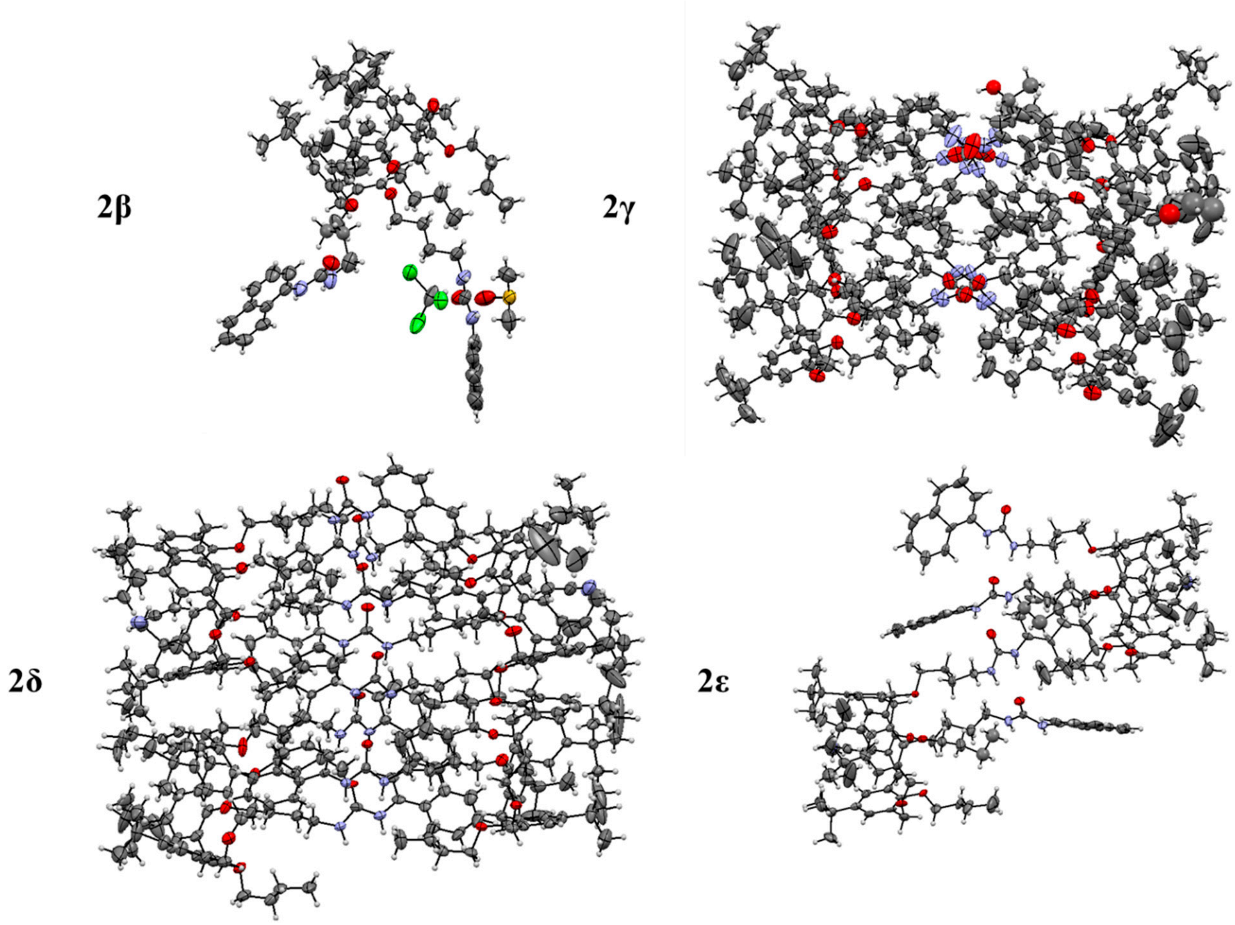

Figure S9. ORTEP drawings (ellipsoids at 50% probability, shown in CPK colours) of the asymmetric units of the four new pseudo-polymorphic forms of compound **2**.

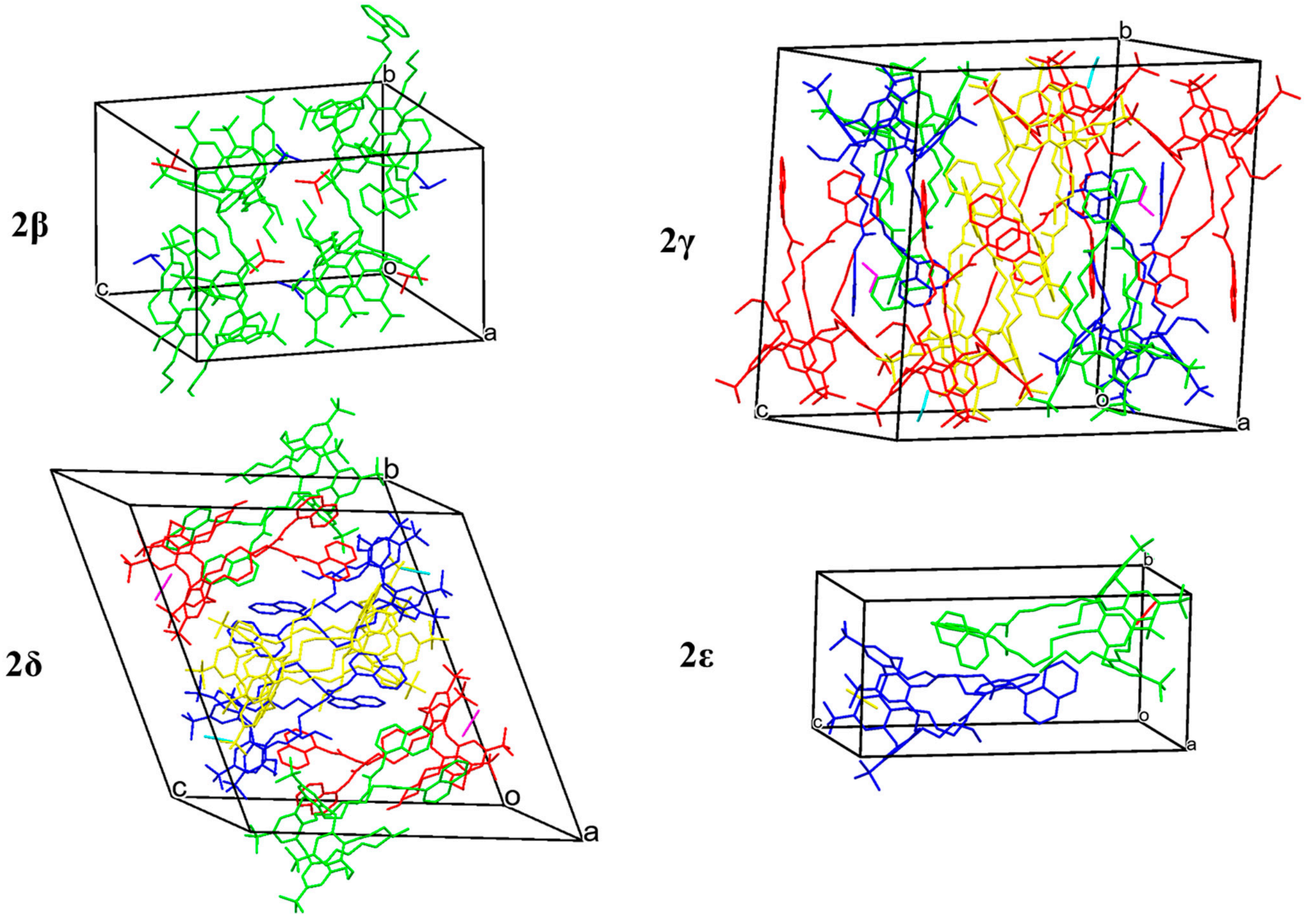

Figure S10. Unit cells of the four new pseudo-polymorphic forms of compound **2**, with symmetry-equivalent molecules shown in the same colour. Hydrogen atoms are omitted for clarity.

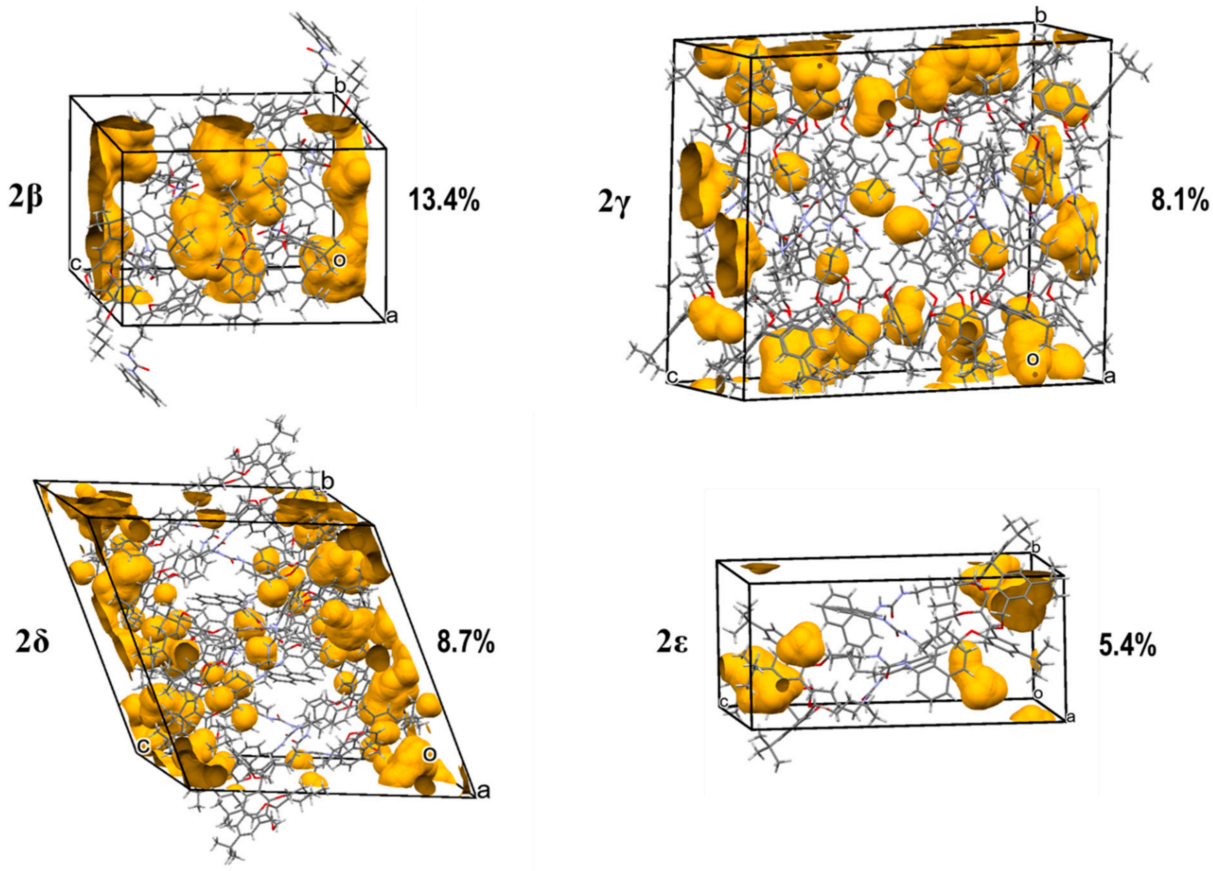

Figure S11. Solvent-accessible volumes of the four new pseudo-polymorphic forms of compound **2**. All co-crystallized solvent molecules were omitted prior to the calculation, which was performed using a solvent probe radius of 1.2 Å.
